# Immune gene expression in the mosquito vector *Culex quinquefasciatus* during an avian malaria infection

**DOI:** 10.1111/mec.16799

**Published:** 2022-12-14

**Authors:** Luz García‐Longoria, Dag Ahrén, Arnaud Berthomieu, Victor Kalbskopf, Ana Rivero, Olof Hellgren

**Affiliations:** ^1^ Department of Anatomy, Cellular Biology and Zoology University of Extremadura Badajoz Spain; ^2^ Molecular Ecology and Evolution Lab, Department of Biology Lund University Lund Sweden; ^3^ MIVEGEC (CNRS, Université de Montpellier, IRD) Montpellier France

**Keywords:** *anopheles*, immunogenicity, immunosenescence, *plasmodium falciparum*, *plasmodium relictum*

## Abstract

*Plasmodium relictum* is the most widespread avian malaria parasite in the world. It is listed as one of the 100 most dangerous invasive species, having been responsible for the extinction of several endemic bird species, and the near‐demise of several others. Here we present the first transcriptomic study focused on the effect of *P. relictum* on the immune system of its vector (the mosquito *Culex quinquefasciatus*) at different times post‐infection. We show that over 50% of immune genes identified as being part of the Toll pathway and 30%–40% of the immune genes identified within the Imd pathway are overexpressed during the critical period spanning the parasite's oocyst and sporozoite formation (8–12 days), revealing the crucial role played by both these pathways in this natural mosquito–*Plasmodium* combination. Comparison of infected mosquitoes with their uninfected counterparts also revealed some unexpected immune RNA expression patterns earlier and later in the infection: significant differences in expression of several immune effectors were observed as early as 30 min after ingestion of the infected blood meal. In addition, in the later stages of the infection (towards the end of the mosquito lifespan), we observed an unexpected increase in immune investment in uninfected, but not in infected, mosquitoes. In conclusion, our work extends the comparative transcriptomic analyses of malaria‐infected mosquitoes beyond human and rodent parasites and provides insights into the degree of conservation of immune pathways and into the selective pressures exerted by *Plasmodium* parasites on their vectors.

## 1 INTRODUCTION

Malaria parasites are one of the most successful parasites on the planet. They are largely known for infecting humans, where they are responsible for an estimated 600,000 deaths per year (World Health Organization, 2021). These protozoans can also be found infecting hundreds of other terrestrial vertebrate species, including nonhuman primates, ungulates, rodents, bats, birds and lizards (Fricke et al., 2010; Schaer et al., 2013; Templeton et al., 2016). There are currently almost a thousand different *Plasmodium* species recognized, with more being described every year. But for a few minor differences, all these species share a nearly identical life cycle, with an asexual replicative stage in the vertebrate host, and a sexual stage in a dipteran vector, usually a mosquito (Eckhoff, 2011; Valkiūnas & Iezhova, 2018).

The importance of mosquitoes in malaria transmission has made them particularly important targets of research (Kar et al., 2014; Keleta et al., 2021; Ryan et al., 2020). Research into the development and testing of new insecticides is ongoing, but the evolution of insecticide resistance has emerged as a serious challenge to malaria control efforts (Moyes et al., 2021). As a result, a great deal of effort is currently focused on alternative strategies aimed at curbing malaria transmission. One such promising approach is the potential to harness mosquito physiology and immunity for the development of transmission‐blocking interventions (Challenger et al., 2021; Wadi et al., 2018). *Plasmodium* parasites go through multiple stages inside mosquitoes. Mosquitoes ingest a bloodmeal containing female and male *Plasmodium* gametocytes, which fuse to become a motile ookinete that traverses the midgut wall to form an oocyst. Oocysts undergo repeated rounds of mitosis to create a syncytial cell with thousands of nuclei. In a massive cytokinesis event, thousands of haploid daughter sporozoites are liberated into the haemolymph, where the infective sporozoites migrate to the mosquito salivary glands for transmission to a new host (Howick et al., 2019; Valkiūnas, 2005).

The mosquito immune system has received considerable attention as mounting evidence points to its critical role in eliminating a sizeable proportion of parasites invading the midgut epithelium (Hajkazemian et al., 2021). In recent years, several genomic and transcriptomic studies performed on the main vectors of human malaria, the *Anopheles gambiae* species complex, have allowed the identification and quantification of numerous transcripts involved in the mosquito immune response to a malaria infection (Carr et al., 2021; Reynolds et al., 2020). There are two main arms of the mosquito innate immune response against *Plasmodium*: the humoral response involving the transcriptional regulation of antimicrobial peptides (AMPs), and the cellular response, which includes phagocytosis and/or melanization (Clayton et al., 2014). Three main signalling pathways have been shown to be involved in the immune cascade that ultimately leads to the destruction of the parasite: the Toll, the immune deficiency (Imd) and the Janus kinase signal transducer of activation (JAK/STAT) pathways (Tikhe & Dimopoulos, 2021). These pathways involve different complex immune cascades that ultimately allow the REL1 (Toll), REL2 (Imd) or STAT (JAK/STAT) transcription factors to enter the nucleus and transcriptionally activate immune effector genes, such as AMPs, complement factors and nitric oxide synthase (NOS) (Clayton et al., 2014). These transcription factors are negatively regulated in the cytoplasm by Cactus (Toll), Caspar and Caudal (Imd), and SOCS and PIAS (JAK/STAT, Clayton et al., 2014).

One key insight from transcriptomic studies has been that natural and artificial mosquito–*Plasmodium* combinations (i.e., species combinations that are found in the wild, or not) have significantly different immune activation profiles (Sreenivasamurthy et al., 2013). For example, rodent *Plasmodium berghei* parasites have a greater impact on the transcriptome of the human malaria vector *An. gambiae*, regulating hundreds of genes belonging to different functional classes, than human *Plasmodium falciparum* parasites (Dong et al., 2006). Most notably, in *An. gambiae* the most effective pathway for the defence against *P. berghei* is the Toll pathway, while for the defence against *P. falciparum* it is the Imd pathway (Clayton et al., 2014; Garver et al., 2009). This suggests that the rodent malaria model may be less relevant to the study of the immune strategies employed by malaria‐infected mosquitoes than a *Plasmodium*–mosquito combination with a long co‐evolutionary history.

Avian malaria has been found infecting thousands of bird species in all geographical regions except Antarctica. Its prevalence and genetic diversity rivals anything that has been found in any other vertebrate malaria (Rivero & Gandon, 2018 but see Bensch et al., 2009). The unparalleled genetic diversity of avian malaria uncovered thus far seems to be matched by an equally rich phenotypic diversity, providing a unique opportunity for exploring the selective pressures under which hosts and parasites evolve (Rivero & Gandon, 2018). In addition, avian malaria has played a key role in the development of knowledge of human malaria parasites, including the elucidation of key aspects of the biology and transmission of *Plasmodium*, and the routine testing and development of the first vaccines and antimalarial drugs (Rivero & Gandon, 2018). Here we present the first transcriptomic analysis of mosquito immune genes in response to a natural avian malaria infection. For this, we use *Plasmodium relictum*, one of the most widespread avian malaria parasites in the world (Kazlauskiene et al., 2013; Valkiūnas et al., 2018). This species has been responsible for several bird species extinctions in Hawaiian birds, as endemic Hawaiian honeycreepers (Drepanidinae genera) (Atkinson & Samuel, 2010; Liao et al., 2017), is threatening several others (most notably in New Zealand and the Galapagos Archipelago: Butchart, 2008; Lapointe et al., 2012) and is currently listed as one of the 100 most dangerous invasive species (Lowe et al., 2000). Its main natural vectors are mosquitoes of the *Culex pipiens* complex, comprising *Culex pipiens* and *Culex quinquefasciatus* (Fonseca et al., 2004). The genome of *C. quinquefasciatus*, which also vectors human filarial parasites and the West Nile virus, was sequenced a decade ago (Arensburger et al., 2010). Since then, the number of studies investigating the transcriptomic response of this species to a pathogen infection has been surprisingly limited (Bartholomay et al., 2010; Girard et al., 2010; Shin et al., 2014) and only one recent study analysed *C. quinquefasciatus* RNA expression during low infection with *P. relictum* with small transcriptional changes observed in mosquito genes related to immune response (Ferreira et al., 2022).

The avian malaria parasite complex consists of a multitude of parasite lineages with most of the lineages showing unique distributions both among hosts species as well as transmission areas (Beadell et al., 2006; Bensch et al., 2009; Hellgren et al., 2015; Valkiūnas, 2005). Some of this variation is thought to be attributed to the immunological compatibility of the vertebrate hosts (Bonneaud et al., 2006; Sepil et al., 2013). In addition to the tolerance and resistance of the vertebrate host possibly affecting the ecological properties of the parasite, the compatibility between the parasite and immune system of different potential vectors might also be part of shaping the same distribution. However, there is a lack of studies on the immunological responses that the vectors exhibit when infected with the parasites within this complex. Here we investigate the temporal gene expression patterns of *C. quinquefasciatus* mosquitoes infected with *P. relictum*, focusing on immune pathways and genes that have been previously identified as being key for the immune response in human vectors of malaria. For this, we compare the transcriptome of infected and control mosquitoes at four different phases of the infection, each corresponding to a key stage of the parasite's development: within the first 30 min of the infected blood meal (gametocyte activation and formation of gametes), and 8 days (peak oocyst production), 12 days (peak sporozoite production) and 22 days (end stages of the infection) later (Valkiūnas, 2005). Full transcriptomic analyses of the parasites at each of these different stages has been published in a separate paper (Sekar et al., 2021). We compare our results to previous work carried out in other mosquito–*Plasmodium* and *Culex*–pathogen combinations. Our study extends the comparative transcriptomic analyses of malaria‐infected mosquitoes beyond human and rodent parasites and provides insights into the degree of conservation of immune pathways and into the selective pressures exerted by *Plasmodium* parasites on their vectors.

## 2 MATERIAL AND METHODS

### 2.1 Experimental design

The aim of the present experiment was to understand how avian malaria infections influence expression patterns of immune genes in mosquitoes at four different key stages of *Plasmodium relictum* development within the mosquito (Sekar et al., 2021): 30 min after the blood meal ingestion (30 min) (gametocyte activation and formation of gametes), 8 days post‐infection (8 dpi) (peak of oocyst production), 12 dpi (peak sporozoite production) and 22 dpi (end stages of the infection). For this, three canaries (Botanic) were infected by injecting them with the blood of a bird infected with *P. relictum* (cytochrome‐b lineage pSGS1) following previously published protocols (Pigeault et al., 2015). The parasitaemia of the source bird was 9%. Three other canaries were used as uninfected controls. The strain of *P. relictum* pSGS1 was collected from the field in 2016 from wild blue tits (*Cyanistes caeruleus*) caught in the region of Montpellier, France, and has been maintained in the laboratory through regular passages between birds (roughly one intraperitoneal passage per month, and one passage through mosquitoes every 3–4 months). *Culex quinquefasciatus* mosquitoes (SLAB strain) have been in culture in the laboratory since their isolation from the San Joaquín Valley (California, USA) in the 1960 s (Georghiou et al., 1966). Mosquito larvae are kept in our insectaries (25–27°C, 70% relative humidity [RH]) at a density of 200 in 30 × 20 × 6‐cm trays filled with 1 L of water and fed a diet of 1/3 Tetramin fish food and 2/3 rabbit pellets.

Ten days later, at the peak of the acute infection stage in blood (Valkiūnas, 2005), the three infected (parasitaemias of 3.55%, 3.05% and 2.85%) and three control birds were placed individually in an experimental cage with 150 7‐day‐old female mosquitoes, which had been reared using standard laboratory protocols (Vézilier et al., 2010). Cages were visited 30 min later to take out the bird and any mosquitoes that were not fully gorged. At this point, 10 fully gorged resting mosquitoes were haphazardly sampled from each of the cages. Although mosquitos could have fed from the bird at any time between 1 and 30 min, the samples were lumped for the analyses (“30 mpi”). Samples were homogenized with 500 μl of TRIzol LS, and frozen at −80°C for subsequent RNA extraction (one pool of 10 mosquitoes per cage). The rest of the mosquitoes were left in their cages with a source of sugar solution (10%) at our standard insectary conditions (25–27°C, 70% RH). Cages were also supplied with a water container to allow egg laying. On day 8 after the blood meal, 10 mosquitoes were randomly taken from each of the three cages (“8 dpi” sample), homogenized with 500 μl of RNAlater and frozen at −80°C. The procedure was repeated on days 12 (“12 dpi” sample) and 22 (“22 dpi” sample). TRIzol was used for the blood‐engorged mosquitoes because bird blood, with its nucleated red blood cells, clogs the filters of the RNA extraction spin columns if they are not first treated in a TRIzol step (see below). To verify the success of the infection, at 8 and 12 dpi, a further sample of 10 mosquitoes per cage was taken and immediately dissected to quantify *Plasmodium* oocysts in the mosquito gut. All the mosquitos analysed harboured parasites.

### 2.2 RNA extraction

All analyses were carried out using pools of 10 mosquitoes (one pool per time point per bird). For the 30 mpi samples, the total volume of the buffer was adjusted to 750 μl; the sample, containing mosquitoes and buffer, was subsequently homogenized using a TissueLyser (Qiagen) equipped with a 5‐mm stainless steel bead. The TissueLyser was run for two cycles of 3 min at 30 Hz. Phase separation was done according to the TRIzol LS manufacturer's protocol; the resulting aqueous phase was mixed with one volume of 70% ethanol and placed in a RNeasy Mini spin column. RNA from the 8, 12 and 22 dpi samples was extracted by first transferring the mosquitoes to a new tube together with 600 μl of buffer RLT and a 5‐mm stainless steel bead and then homogenized using a TissueLyser. The TissueLyser was run for two cycles of 3 min at 30 Hz. Afterwards RNA was extracted using RNeasy Mini spin columns following the manufacturer's protocol.

The concentration of all RNA samples was measured on a Nanodrop 2000/2000c (Thermo Fisher Scientific). mRNA from each time point was sequenced using an Illumina HiSeq platform at an average of 85 million reads per library (Novogene). We obtained paired‐end reads 150 bp in length.

### 2.3 Data processing

Sequence data quality testing was performed using fastqc (version 0.11.8) (Andrews, 2020). Low‐quality reads were filtered or trimmed with trimmomatic (version 0.27) (Bolger et al., 2014). The resulting files were aligned with star (version 2.7.9a) (Dobin et al., 2013) by using the *C. quinquefasciatus* genome as reference (Holt et al., 2002). Finally, read count per gene was performed using featurecounts (version 2.0.1.1) (Liao et al., 2014).

### 2.4 Statistical analyses

All the statistical analyses were carried out with the free statistical software R (R Core Team, 2020) and the free integrated development environment rstudio (Rstudio Team, 2020). The package deseq2 (version 1.16.1) (Love et al., 2014) was used to estimate the variance–mean dependence in count data from high‐throughput sequencing assays and to test for differential expression based on a model using the negative binomial distribution. When testing for significant differences in expression, and to avoid problems arising from sequencing depth, gene length or RNA composition, the count data were first normalized in deseq2 (Bushel et al., 2020). Principal component analyses (PCAs) were built with a deseq2 function for plotting PCA plots, which uses ggplot2 (Wilkinson, 2011).

For the purpose of investigating how different immune pathways are activated in the mosquito during the infection, a list of genes was generated for each pathway through the Vector Base bioinformatic resource (Amos et al., 2021). We also selected genes that have an important role in the melanization cascade, notably genes involved in proteolytic activation of the phenoloxidase cascade: prophenoloxidase (proPO), serines proteases and their serpin inhibitors (Zhang et al., 2016).

At each time point, the expression levels of the immune genes were compared between infected and control mosquitoes. For these analyses, we used a differential expression analysis based on the Negative Binomial (a.k.a. Gamma‐Poisson) distribution though the function DESeq in the deseq2 package (Bushel et al., 2020).

To further investigate the drastic decrease in differential expression of all gene pathways towards the end of the infection (day 22), we analysed the differences in gene expression occurring across the different time transitions (30 mpi to 8 dpi, 8 dpi to 12 dpi, and 12 dpi to 22 dpi) in infected and control mosquitoes. This analysis did not distinguish between immune pathways (i.e., all the immune genes were lumped together irrespective of their pathway). These analyses were also carried out using the function DESeq (see above). In these analyses, genes differing in their expression levels with a adjusted *p* < .05 were considered to be differentially expressed.

Statistical analysis of the raw TPM (transcripts per kilobase million) values across time points for 23 genes found to be overexpressed during the last time point was carried out using a mixed model (lmer procedure within the lme4 library) fitting infection status (infected, noninfected) and sampling time as fixed explanatory variables and birds as a random variable. The significance of the explanatory variables was established using a likelihood ratio test (LRT) which is approximately distributed as a chi‐square distribution and using *p* = .05 as a cut‐off *p*‐value.

## 3 RESULTS

### 3.1 Principal component analysis

**FIGURE 1:**
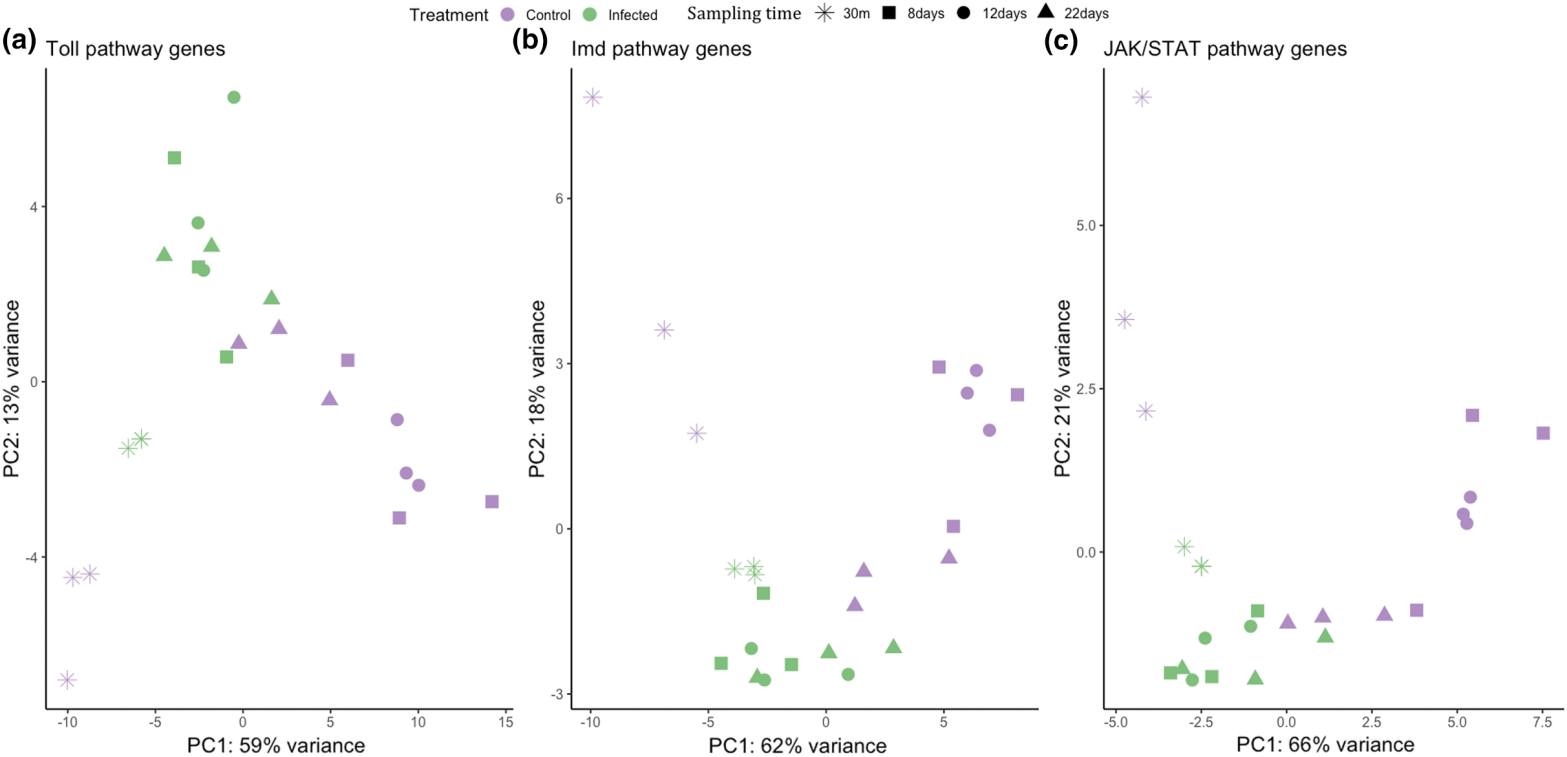
Principal component analyses of expression levels from the three immune pathways (a: Toll, b: Imd, c: JAK/STAT). Results have been colour coded depending on the mosquito condition: Green infected; purple control. Different sampling times are shown with different shapes

A PCA was run for each group of genes (Toll, Imd and JAK/STAT pathways; Figure 1). The first (PC1) and second (PC2) axes explain 59% and 13% of variance in the expression of genes of the Toll pathway (Figure 1a), 62% and 18% of the Imd pathway (Figure 1b), and 66% and 21% of the JAK/STAT pathway (Figure 1c). Based on visual evaluation of Figure 1, the PCAs show differences in the global expression patterns of infected and noninfected mosquitoes across the four different time points in the three immune pathways. The largest differences in scores across both PC1 and PC2 are seen in control mosquitoes at 30 mpi. A certain discrimination based on scores of PC was also observed in control mosquitoes at 8 and 12 dpi. Interestingly, there was no clear discrimination between the expression levels of infected mosquitoes across different sampling times, except for genes in the Toll pathway of 30 mpi mosquitoes which cluster separately from their control counterparts (Figure 1a).

### 3.2 Expression pattern of genes in the three immune pathways

**FIGURE 2:**
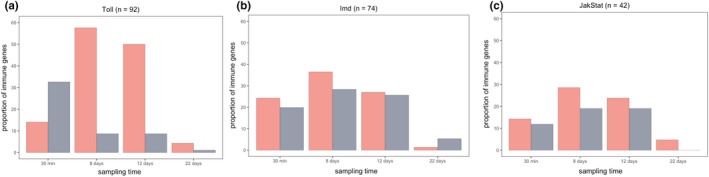
Proportion of differentially expressed genes (DEGs) that are up‐regulated (pink) or down‐regulated (grey) at each sampling point after infection with *plasmodium relictum*. The name of the pathway and total number of genes identified for each pathway are given in the header

Our analyses included a total of 92 immune genes belonging to the Toll pathway, 74 to the Imd pathway and 42 to the JAK/STAT pathway. The number of differentially expressed genes (DEGs) differed widely between pathways and sampling points (Figure 2—see below). In particular, there is a striking up‐regulation of Toll pathway genes at 8 and 12 dpi, and a near absence of DEGs towards the end of the infection (22 dpi).

We classed the DEGs into four different functional classes: receptors, transcription factors, inhibitors and effectors, and the magnitude of gene expression differences between infected and uninfected mosquitoes was represented as log‐fold change (LFC) values (Figure 2). The highest overall LFC values are observed within the Toll pathway. Many of the receptors, transcription factors and effectors within the Toll and Imd pathways followed a similar pattern: they were higher in infected mosquitos at 8 and 12 dpi than earlier (30 mpi) and later (22 dpi) in the infection (Figure 2; but also see Table S1). This pattern was, however, less apparent within the JAK/STAT pathway (Figure 2c). Four of the seven inhibitor genes identified were under‐expressed while two others (one Imd and one JAK/STAT) were significantly over‐expressed at 8 and 12 dpi (Figure 2).

### 3.3 Biological function of differentially expressed genes

**FIGURE 3:**
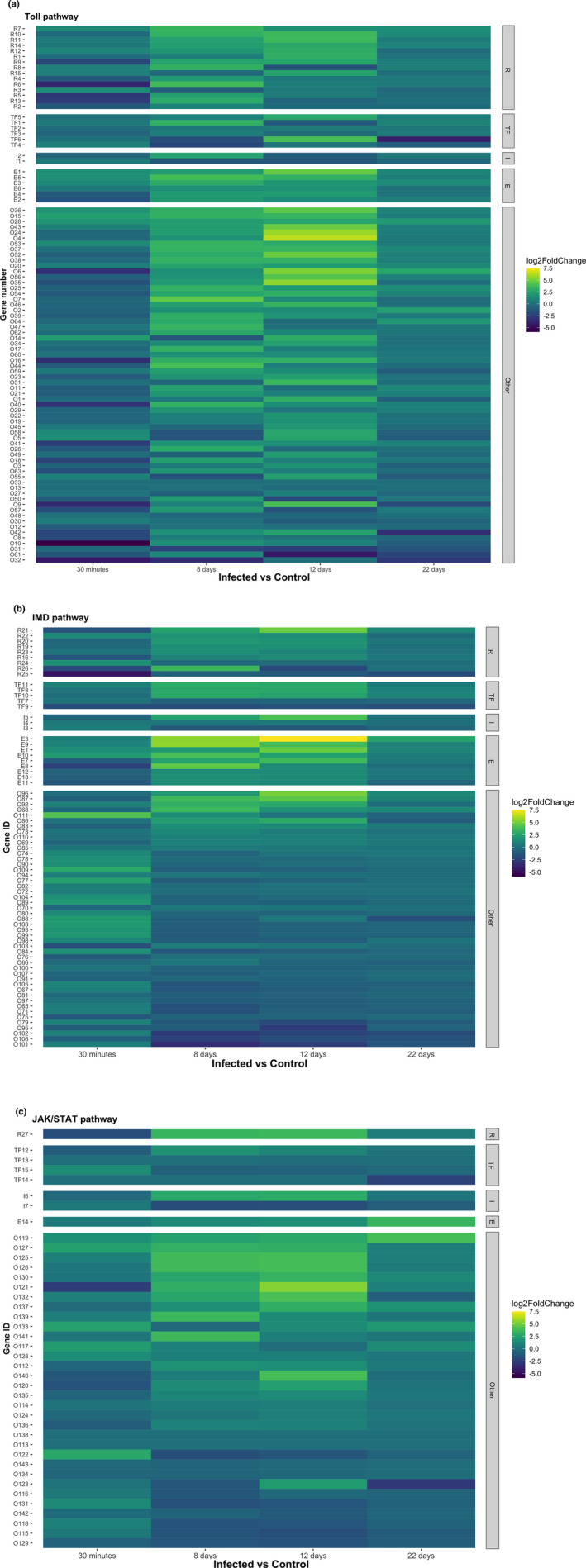
Heatmaps for the different pathways showing log‐fold change (LFC) values resulting from the statistical comparison of infected vs. control mosquitos at each sampling time (a: Toll, b: Imd, c: JAK/STAT). LFC values were classified as follows: Receptors (“R”), transcription factors (“TF”), inhibitors (“I”), effectors (“E”) and “other” (grouping all other genes within the pathway)

**FIGURE 4:**
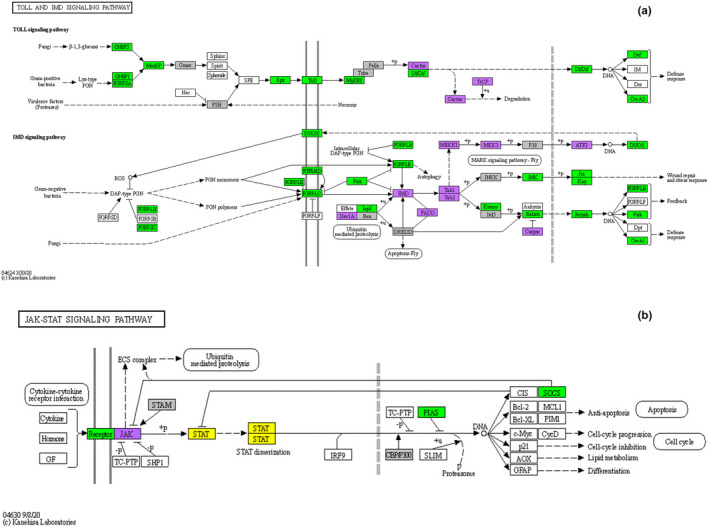
Differentially expressed genes (DEGs) at 8 dpi (peak oocyst) and 12 dpi (peak sporozoite) mapped onto the KEGG toll and Imd signalling pathways of *Drosophila melanogaster* (Kanehisa et al., 2021) (a), and JAK/STAT pathway adapted from the *Homo sapiens sapiens* pathway (Kanehisa et al., 2021) (b). Green: Genes up‐regulated in infected mosquitoes as compared to control mosquitoes. Purple: Genes down‐regulated. Grey: no significant difference in expression but with observed expression in the data. White: Genes not present in our analysis. Yellow: Genes detected but with zero transcripts (TPM) at all sampling times. Genes with N/a values are not plotted (see Tables S1 and S2 for corresponding gene names)

We identified a significant up‐regulation of genes related to the Imd and Toll pathways (Figure 3a; Table S2). Most of the genes present in the Toll and Imd pathways showed either significant up‐ or down‐regulation during the different infection phases. For the JAK/STAT pathway most of the genes detected were either down‐regulated or not differentially expressed (Figure 3b; Table S2). The biological function of the genes within the Imd, Toll and JAK/STAT pathways are shown in Figure 4. The pattern of up‐ or down‐regulation of these different genes are discussed below separately for each sampling time.

#### 3.3.1 Thirty minutes post‐blood meal

**FIGURE 5:**
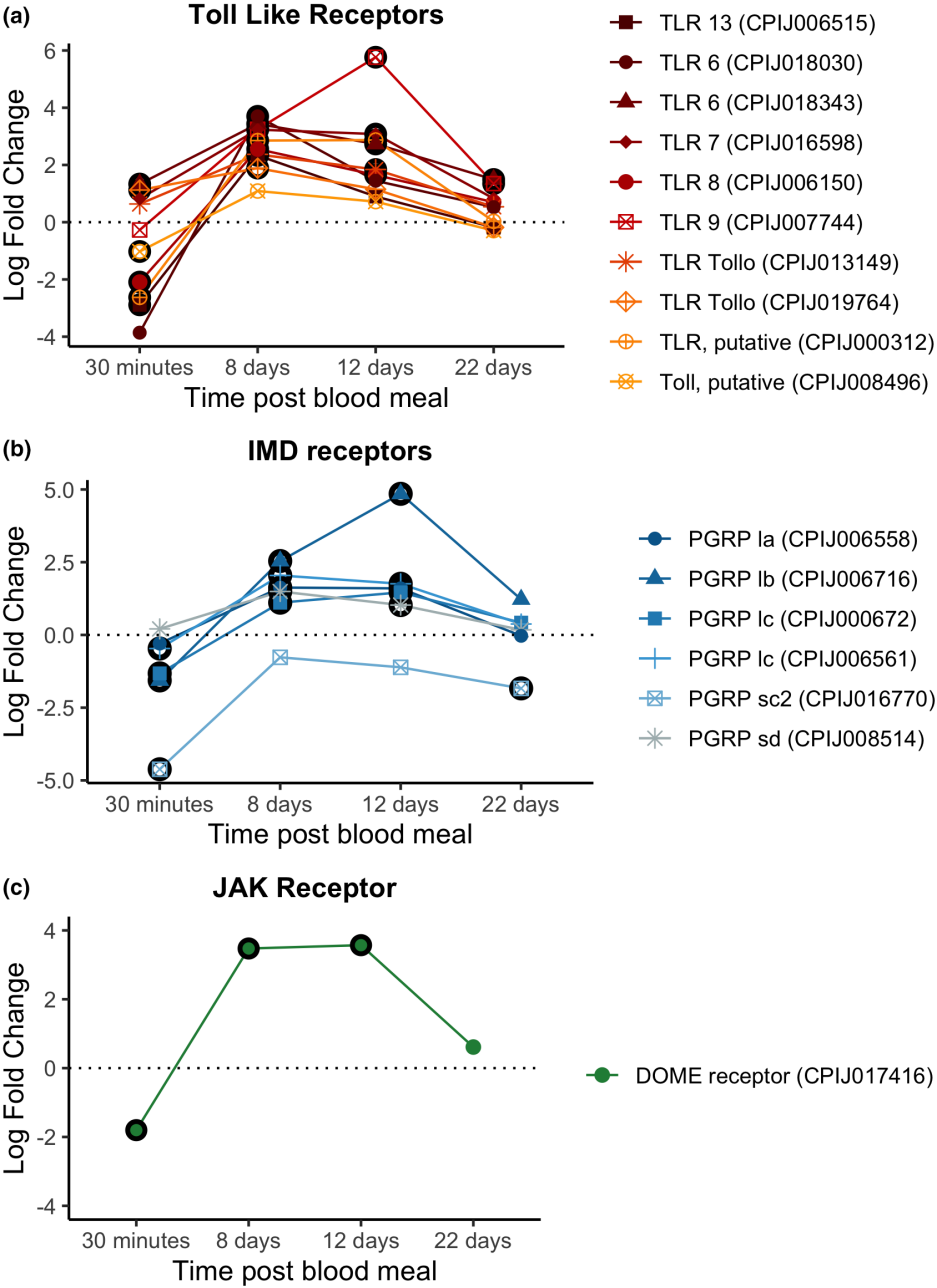
Differential expression pattern of receptor genes between infected and control mosquitoes across different sampling points. Differential expression is represented as log‐fold change (LFC) values. (a) Toll pathway, (b) Imd pathway, (c) JAK/STATS pathway. Values above the dashed horizontal line indicate a higher expression in infected than uninfected mosquitoes. The black circle around each dot indicates a significant difference when comparing infected and control mosquitos (adjusted *p* < .05)

Only two Toll receptors (CPIJ019764, CPIJ018343, Figure 5a) were significantly up‐regulated at 30 mpi.

**FIGURE 6:**
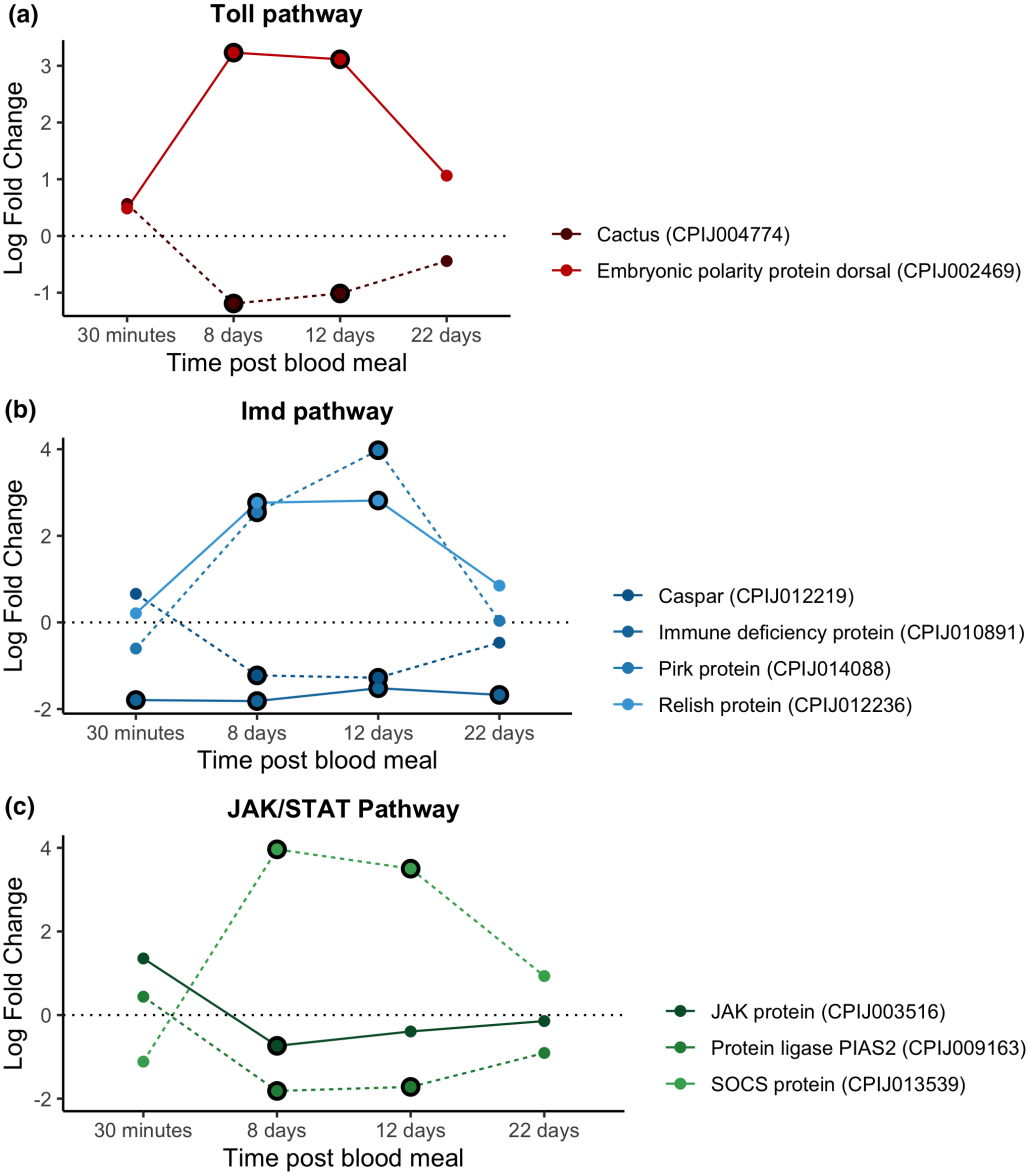
Differential expression pattern of transcription factor and inhibitor genes between infected and control mosquitoes across different sampling points. Differential expression is represented as log‐fold change (LFC) values. (a) Toll pathway, (b) Imd pathway, (c) JAK/STAT pathway. Each colour represents a transcription factor or inhibitor combination. Dark tones and continuous lines indicate transcription factors and soft tones and dashed lines indicate inhibitor genes. Note that each transcription factor has the same colour as its inhibitor (blue, orange or purple). The black circle around each dot indicates a significant difference when comparing infected and control mosquitoes (adjusted *p* < .05)

**FIGURE 7:**
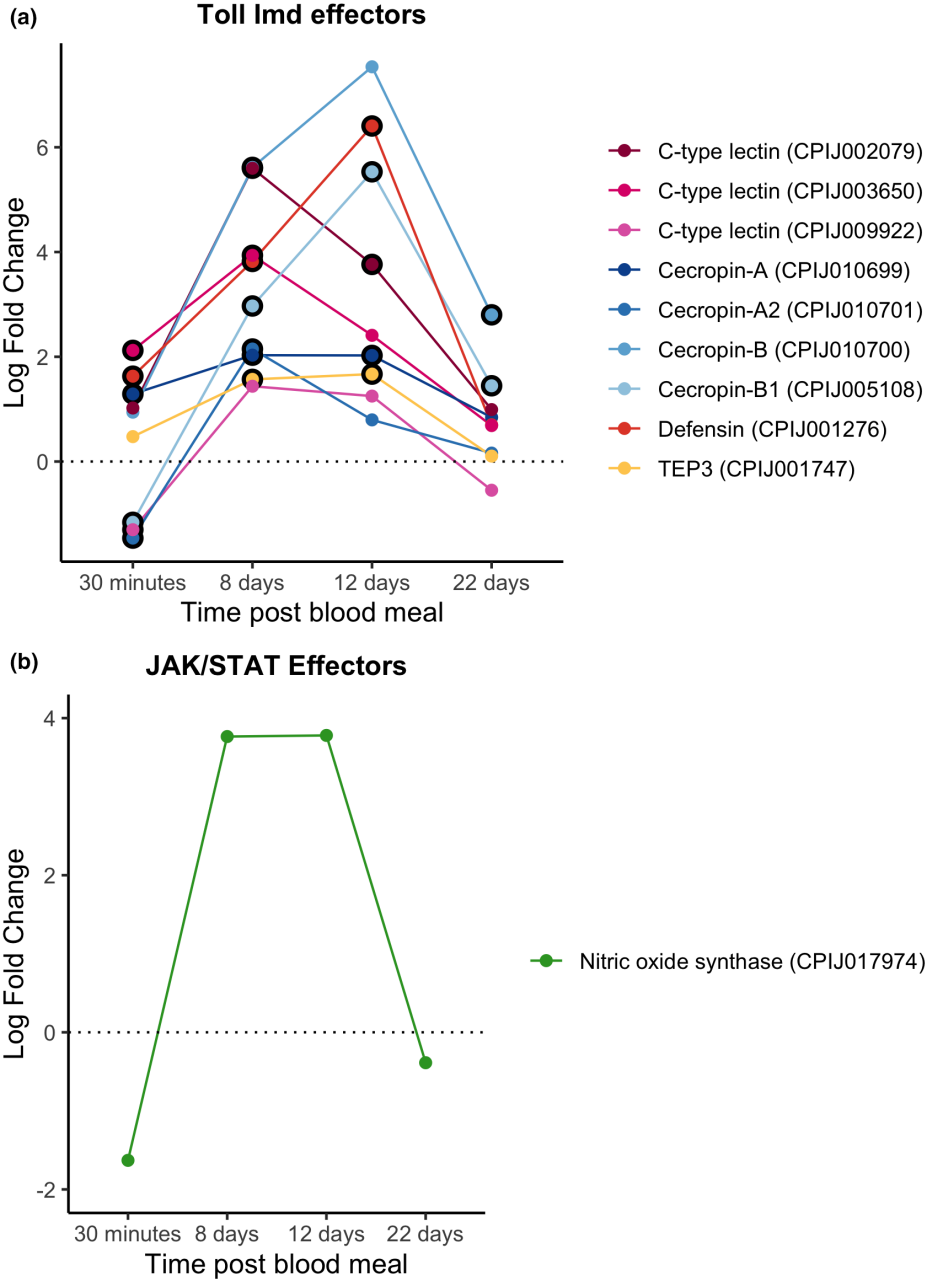
Differential expression pattern of effector genes between infected and control mosquitoes across different sampling times. Differential expression is represented as log‐fold change (LFC) values. (a) Effectors for the toll and Imd pathways; (b) NOS. The black circle around each dot indicates a significant difference when comparing infected and control mosquitos (adjusted *p* < .05)

We did not detect any up‐regulated transcription factors in either the Toll, Imd or JAK/STAT pathways (Figure 6). In contrast, there was a down‐regulation of the Imd transcription factor (CPIJ010891) in infected mosquitos (Figure 6b). No inhibitors were significantly up‐ or down‐regulated for any of the three pathways at this time point. Interestingly, some effectors such as defensin‐A and cecropin‐A were up‐regulated as early as 30 mpi, while others such as cecropin genes (A2 and B1) were significantly down‐regulated (Figure 7a). We did not detect any significant difference in the expression of NOS (Figure 7b).

**FIGURE 8:**
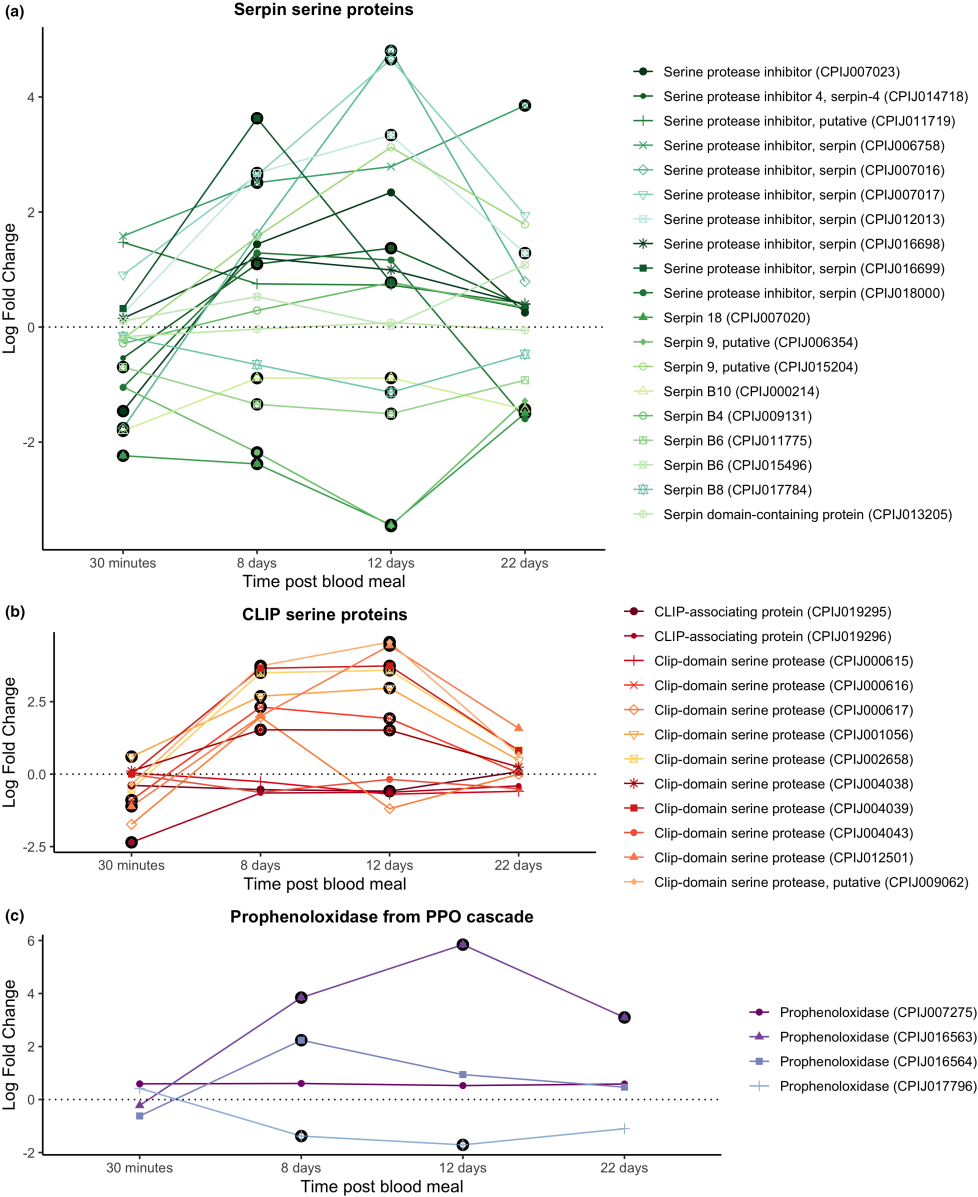
Differential expression pattern of (a) serpin serine proteins, (b) CLIP serine proteins and (c) prophenoloxidase from the PPO cascade. Differential expression is represented as log‐fold change (LFC) values. The black circle around each dot indicates a significant difference when comparing infected and control mosquitos (adjusted *p* < .05).

Genes of the PPO cascade were not significantly up‐regulated at this sampling time (Figure 8).

#### 3.3.2 Eight and 12 days post‐blood meal

The 8 dpi time point is associated with a significant up‐regulation of receptors in the Toll, Imd and JAK/STAT pathways (Figure 5). The level of expression of these receptors did not change significantly between 8 and 12 dpi, except for TLR9 (CPIJ007744) in the Toll pathway and PRPlb (CPIJ006716), which showed a further increase.

The Dorsal transcription factor within the Toll pathway (CPIJ002469) was differentially up‐regulated at 8 and 12 dpi while its inhibitor, cactus protein (CPIJ004774), was significantly down‐regulated (Figure 6a). A similar pattern was observed with the relish transcription factor (CPIJ012236) and its caspar inhibitor (CPIJ012219) within the Imd pathway (Figure 6b). In contrast, an opposite pattern (down‐regulation of the transcription factor, up‐regulation of the corresponding inhibitor) was observed for the Immune deficiency protein transcription factor (CPIJ010891) and Pirk inhibitor (CPIJ014088) in the Imd pathway (Figure 6b), and the Signal transducing adapter molecule 1 (CPIJ003516) and its SOCS inhibitor (CPIJ013539, Figure 6c).

As expected, most antimicrobial peptides and effectors were significantly up‐regulated at 8 and 12 dpi (Figure 7a). NOS showed a similar pattern, with a drastic increase between 30 mpi and 8 dpi, even though the differential expression levels did not reach statistical significance at any of the time points (Figure 7b).

CLIP serine proteases and prophenoloxidases from the PPO cascade were significantly up‐regulated at this sampling time (Figure 8) while some serpin proteins remained significantly down‐regulated (Figure 8c).

#### 3.3.3 Twenty‐two days post‐blood meal

Receptor expression decreased drastically between the 12 and 22 dpi time points. Two Toll receptors (CPIJ018343 and CPIJ007744) were significantly up‐regulated at 22 dpi (Figure 5a). One of the receptors in the Imd pathway (CPIJ016770), however, was significantly down‐regulated.

Neither the Toll transcription factor nor its inhibitor protein were differentially expressed at 22 dpi (Figure 6a). The same results were observed for Relish and Caspar within the Imd pathway (Figure 6b). The Immune deficiency transcription factor was significantly down‐regulated in infected mosquitoes throughout the course of the infection (Figure 6b).

Antimicrobial peptide and NOS expression decreased drastically between the 12 and 22 dpi time points. Only cecropin‐B and B1 (CPIJ0005108, CPIJ010700) were significantly up‐regulated throughout the infection (Figure 7a).

No CLIP serine proteins or prophenoloxidases were significantly up‐regulated except for one prophenoloxidase (CPIJ007275) (Figure 8). Most serpin genes were not significantly expressed (Figure 8a).

**FIGURE 9:**
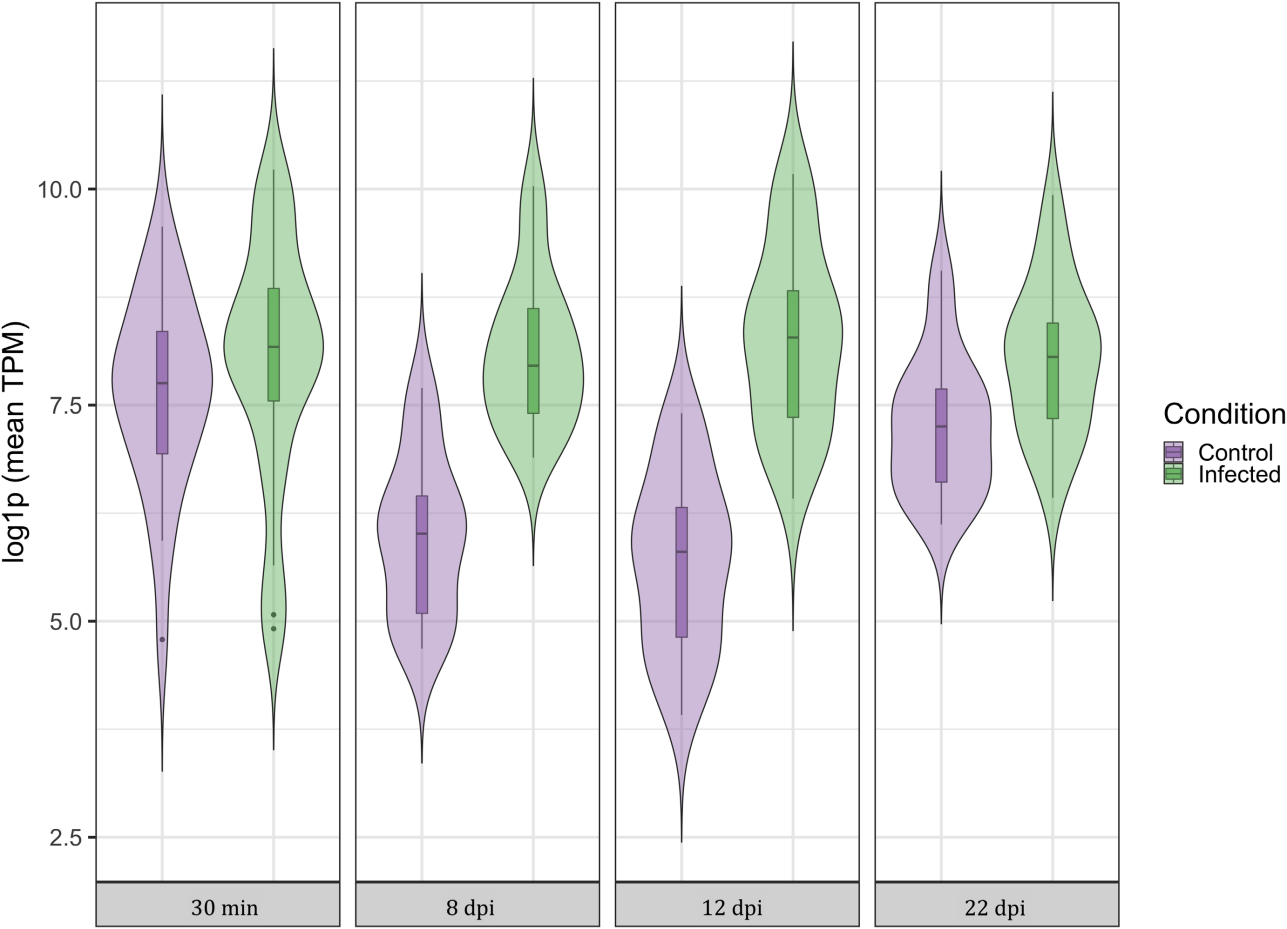
Violin plot of TPM (transcripts per kilobase million) values for 23 immune genes that were up‐regulated at the end of the experiment in control mosquitos compared to infected mosquitoes (see Figures S1 and S2). Violins represent both control and infected mosquitos at each of the different sampling times (30 min, 8 dpi, 12 dpi and 22 dpi). TPM values have been log‐transformed to normalize the plot.

Our results showed that the lack of differential expression between infected and uninfected mosquitoes was due to an increase in expression in uninfected mosquitoes rather than a decrease in expression in infected ones (Figure S1). Uninfected mosquitoes had a set of 23 genes which were upregulated between 12 and 22 dpi (Figure S1 and Table S3). Analyses of the raw TPM values across infection status (infected, control) and sampling times of these 23 genes confirmed that while in infected mosquitos these genes maintained the same level of expression between 30 mpi and 22 dpi, in control mosquitos expression decreased significantly at 8 and 12 dpi only to increase again at 22 dpi (χ^2^ = 88.47, *p* < .0001, Figure 9; see also Figures S1 and S2).

## 4 DISCUSSION

*Culex quinquefasciatus* is an epidemiologically important vector of an exceptionally diverse array of taxonomically different pathogens, including arboviruses (West Nile, St. Louis encephalitis and Rift Valley viruses), filarial worms (*Wuchereria bancrofti*) and protozoans (avian *Plasmodium* and avian *Trypanosoma*). Although *Culex* mosquitoes diverged from their *Anopheles* and *Aedes* counterparts during the early Jurassic (~160–200 million years ago) and early Cretaceous (~130 million years ago) periods, respectively (da Silva et al., 2020; Lorenz et al., 2021), they still share pathogen groups for which they act as vectors for, for example, *Plasmodium* spp. The first *C. quinquefasciatus* genome sequence revealed a significant expansion in the number of immune genes compared with *Anopheles gambiae* (+120 genes) and *Aedes aegypti* (+83 genes (Bartholomay et al., 2010), possibly reflecting the higher diversity of pathogens to which this species is exposed both during its larval and adult lives. While in the last decade the number of transcriptomic studies identifying the immune pathways activated in *Anopheles* and *Aedes* mosquitoes in response to malaria and arboviral infections has grown exponentially (Cirimotich et al., 2010; Ruiz et al., 2019; Singh et al., 2021; Vargas et al., 2020), we still know comparatively little about how *Culex* mosquitoes respond to infections; but see Girard et al., 2010; Ferreira et al., 2022).

Transcriptomic analyses of *C. quinquefasciatus* infected with *Plasmodium relictum*, a highly prevalent (Hellgren et al., 2015) and often virulent (Bueno et al., 2010; Cellier‐Holzem et al., 2010) avian malaria parasite, reveal dynamic changes in genes in the Toll, Imd and, to a lesser extent, JAK/STAT immune pathways throughout the infection. Previous work on the key human malaria vector, *Anopheles gambiae*, has shown that while the Toll pathway is particularly effective against rodent *Plasmodium berghei* parasites, human *Plasmodium falciparum* parasites are largely controlled via the Imd pathway (Clayton et al., 2014). The reasons for this are not yet clear, although these striking differences in immune response have sounded a cautionary note about the dangers of interpreting the transcriptomes of non‐natural mosquito–*Plasmodium* combinations (Boëte, 2005).

Here we show that in *C. quinquefasciatus* infected with *P. relictum*, over 50% of immune genes identified as being part of the Toll pathway and 30%–40% of the immune genes identified within the Imd pathway are overexpressed during the critical period spanning oocyst and sporozoite formation (8–12 dpi), revealing the crucial role played by both these pathways in natural mosquito–*Plasmodium* combinations. A significant up‐regulation of Toll‐like receptors (TLRs) and Peptidoglycan recognition receptors (PGRRs) is observed. In addition, key transcription factors within the Toll (Dorsal) and Imd (Relish) pathways are significantly more highly expressed in infected vs. control mosquitoes, while their corresponding inhibitors (Cactus and Caspar, respectively) are significantly less expressed. A wide range of genes controlling immune effectors are also significantly more expressed in infected vs. control mosquitoes at 8–12 dpi, including several genes coding for antimicrobial peptides which have been previously shown to play an important role in the clearance of rodent and human *Plasmodium* (Cecropin A, Cecropin B and Defensin; Kokoza et al., 2010, Raulf et al., 2019, Simões et al., 2022). NOS, the enzyme responsible for the production of nitric oxide, which has been shown to be linked to *Plasmodium* killing in the midgut (Kumar et al., 2003; Luckhart et al., 1998; Peterson et al., 2007), was also overexpressed in infected relative to uninfected mosquitoes although the differences were not statistically significant.

*Plasmodium* oocysts are vulnerable to melanization, a cascade that initiates with the proteolytic activation of proPO and which is controlled by a series of CLIP‐domain serine proteases and their serpin inhibitors (Zhang et al., 2016). Two CLIP serine proteases, CLIPB8 and CLIPB9, and a one serpin, SRPN2, have been identified as crucial elements of the melanization cascade in *An. gambiae* mosquitoes infected with human malaria. Several other CLIPB proteinases, including CLIPB1, 3, 4, 14 and 17, also affect rodent malaria parasites (Cao et al., 2017). At 8–12 dpi we observed an over‐expression of several proPOs and CLIP‐domain serine proteases, and an under‐expression of serpins, indicating that the phenoloxidase (melanization) cascade may play an important role in the defence against *P. relictum*. At this point, we do not know which, if any, of the CLIPs and serpins triggered in *Culex pipiens* in response to a *Plasmodium* infection play a role in the mosquito melanization cascade.

An unexpected result was the significant differences in expression of several immune effectors as early as 30 mpi. Although the exact time course of the onset of an avian malaria infection in mosquitoes has not yet been entirely elucidated (Rivero & Gandon, 2018), extrapolations from human and rodent malaria would suggest that at this time point the gametocytes ingested with the blood meal are transforming to gametes before fusing to produce a motile zygote within the blood bolus (Dong et al., 2006; Tahar et al., 2002). Several immune effectors (Cecropin A, Defensin and C‐type lectin) are significantly over‐expressed in infected mosquitoes at this early time point. Several nonexclusive explanations can be proposed for this observed phenomenon. First, gametes or zygotes may be immunogenic. Several *Plasmodium* proteins (e.g., P25/P28), which are transcriptionally repressed in the gametocyte, are expressed as soon as the gametes are formed (del Carmen Rodriguez et al., 2000). These proteins are highly immunogenic for the vertebrate immune system (Saxena et al., 2007), but whether they may also trigger an immune response in mosquitoes is currently unknown. In a companion paper investigating *P. relictum* gene expression in this same experiment (Sekar et al., 2021), high expression of P25 was found both in bird blood (immediately before the mosquito blood meal) and within the mosquito at 30 mpi, but very low expression at later time points (see Sekar et al., 2021; gene PRELSG_0614600). Second, the mosquito may (also) be reacting to the plasma of infected hosts. Plasma from *Plasmodium*‐infected hosts contains several components of the host's immune response (e.g. IgG, IgE and IgM antibodies, and TNF‐α and related inflammatory cytokines) as well as extracellular vesicles shuttling communication signals between parasites during an infection (Clark, 2007; Opadokun & Rohrbach, 2021). When injected to a new vertebrate host, plasma from *Plasmodium*‐infected individuals can exert powerful immune responses (Couper et al., 2010), though whether it can trigger a similar response in mosquitoes remains to be tested. Several other immune components (e.g., Cecropin B) were, in contrast, significantly down‐regulated at 30 mpi, a phenomenon that suggests an immunomodulatory effect of the early stages of the parasite's infection in the mosquito. *P. falciparum* Pfs47, which is expressed in gametes and early ookinetes, actively supresses a key immune signalling cascade that ultimately leads to the TEP1‐mediated lysis of the parasite (Molina‐Cruz et al., 2013, 2020; Ramphul et al., 2015). In *P. relictum*, a Pfs47 orthologue was found to have high expression levels in bird blood as well as in the mosquito at 30 mpi, with nearly nonexistent expression at later time points (see Sekar et al., 2021; gene PRELSG_1251100).

Towards the end of the infection (22 dpi) the differences in immune gene expression between infected and uninfected mosquitoes are drastically reduced. Time transition analyses revealed that the lack of differences in both Toll and Imd pathways towards the end of the mosquito lifespan was due to an increase in immune gene expression in control mosquitoes rather than to a decrease in infected ones (Figure S2; Figure 9). This increase in immune investment towards the end of the mosquito life is paradoxical, as the general expectation is for a decline in immune function with age (immune senescence) as a result of physiological wear and tear, or of an adaptive reallocation of immune resources towards other traits such as reproduction or longevity (Shanley et al., 2009). Our results contrast with previous results obtained in the *C. pipiens*/*P. relictum* system, which showed a decrease in PO activity (Cornet & Sorci, 2010) and haematocyte counts (Pigeault et al., 2015) with age, as well as a higher susceptibility to a *P. relictum* infection in old mosquitoes (Pigeault et al., 2015). An increase in immune gene transcripts with age has, however, also been observed in *Drosophila melanogaster* (Zerofsky et al., 2005), which has been tentatively interpreted as the result of an age‐related failure of one of the myriad mechanisms responsible for regulating the immune response (Zerofsky et al., 2005), highlighting the limitations of extrapolating immune function from immune transcript quantification.

In conclusion, these results highlight the similarities between the immune pathways activated by *P. relictum* in *C. quinquefasciatus* mosquitoes and those activated by *P. falciparum* in *An. gambiae* mosquitoes. There are also some key differences: while *An. gambiae* predominantly uses the Imd immune pathway to fight a *P. falciparum* infection (Clayton et al., 2014), both the Imd and Toll pathways seem to be essential for *C. quinquefasciatus* during a *P. relictum* infection. One potential caveat of this study is that by doing the transcriptomic analysis on whole mosquitoes, some of the tissue‐specific immune signals may have been omitted or diluted within the general transcriptomic “noise.” Future studies should carry out tissue‐specific transcriptomics (midgut for the oocyst stage, salivary glands for the sporozoite stage) which would provide a more targeted picture of the immune pathways used by the mosquito in its fight against *Plasmodium*.

By comparing infected and uninfected mosquitoes at different time points after the bloodmeal we also revealed a dynamic pattern of gene expression that raises interesting questions about the onset of *Plasmodium* immunogenicity and mosquito immunosenescence, showcasing the added value of temporal transcriptomic studies of *Plasmodium*–mosquito interactions. It is worth pointing out the differences between our results and those of a recent study looking at the transcriptomics of *C. pipiens* mosquitoes infected with a different cytochrome b‐lineage of *P. relictum* collected from Hawaii's ‘amakihi honeycreepers (pGRW4; Ferreira et al., 2022). This previous study surprisingly found no over‐expression of immune genes in infected mosquitoes at any time point (24 h to 10 days post‐blood feeding). Whether this is due to the different cyt‐b lineages used or to differences in the experimental protocols is unknown. Although pSGS1 and pGRW4 are currently grouped within the same *P. relictum* morphospecies, previous work has revealed a significant phylogenetic difference between these two lineages (Hellgren et al., 2015). The extraordinary diversity of avian *Plasmodium* parasites (Bensch et al., 2009) offers an unparalleled opportunity to explore the specificity of the mosquito's immune system.

Avian malaria has been at the forefront of research into bird conservation since the accidental introduction of *P. relictum* to the Hawaiian Islands in the early twentieth century, which resulted in the local extinction of several native bird species (Atkinson et al., 2000). In the ensuing years, avian malaria has been introduced into other regions such as the Galapagos Islands and New Zealand, raising concerns that spread into the endemic avifauna of these regions might parallel what happened to native Hawaiian forest birds (Levin et al., 2009). Because malaria dynamics are strongly influenced by ambient temperature and precipitation patterns, predicted future climate is expected to increase the occurrence, distribution and intensity of avian malaria transmission to new areas. As in the case of human malaria (Dong et al., 2020), the most promising approach for limiting the effect of avian malaria on wild bird populations is through interventions aimed at limiting transmission by the mosquitoes. Knowledge of the immune responses that mosquitoes mount in response to an avian malaria infection is a crucial first step towards the implementation of soon to be available tools such as mosquito transgenesis. By introducing one of the first studies on the immunological pathways for avian malaria vectors during infection, we further hope to provide initial knowledge for future studies on how the immunological compatibility between the parasite and the vectors might contribute to the spatial and the host distributions observed in the wild.

## AUTHOR CONTRIBUTIONS

OH, AR and AB conceptualized and designed the methodology for the whole experiment. LGL carried out data curation, formal analyses and writing of the original draft. OH and AR review and editing the final manuscript and provided supervision. DA and VK supervised the writing process and helped with data curation, formal analyses and software problems.

## CONFLICT OF INTEREST

The authors declare that they have no conflicts of interest.

## Supporting information


Figure S1

Figure S2

Table S1

Table S2

Table S3

## Data Availability

Sequences have been uploaded to the Sequence Read Archive (SRA) at NCBI under accession no.: PRJNA848963.
