# Supplementary figures and images for: Immune gene expression in the mosquito vector Culex quinquefasciatus during an avian malaria infection
Source: Mol Ecol. 2022 Dec 14;32(4):904–19. doi: 10.1111/mec.16799 (PMC10108303; doi:10.1111/mec.16799)

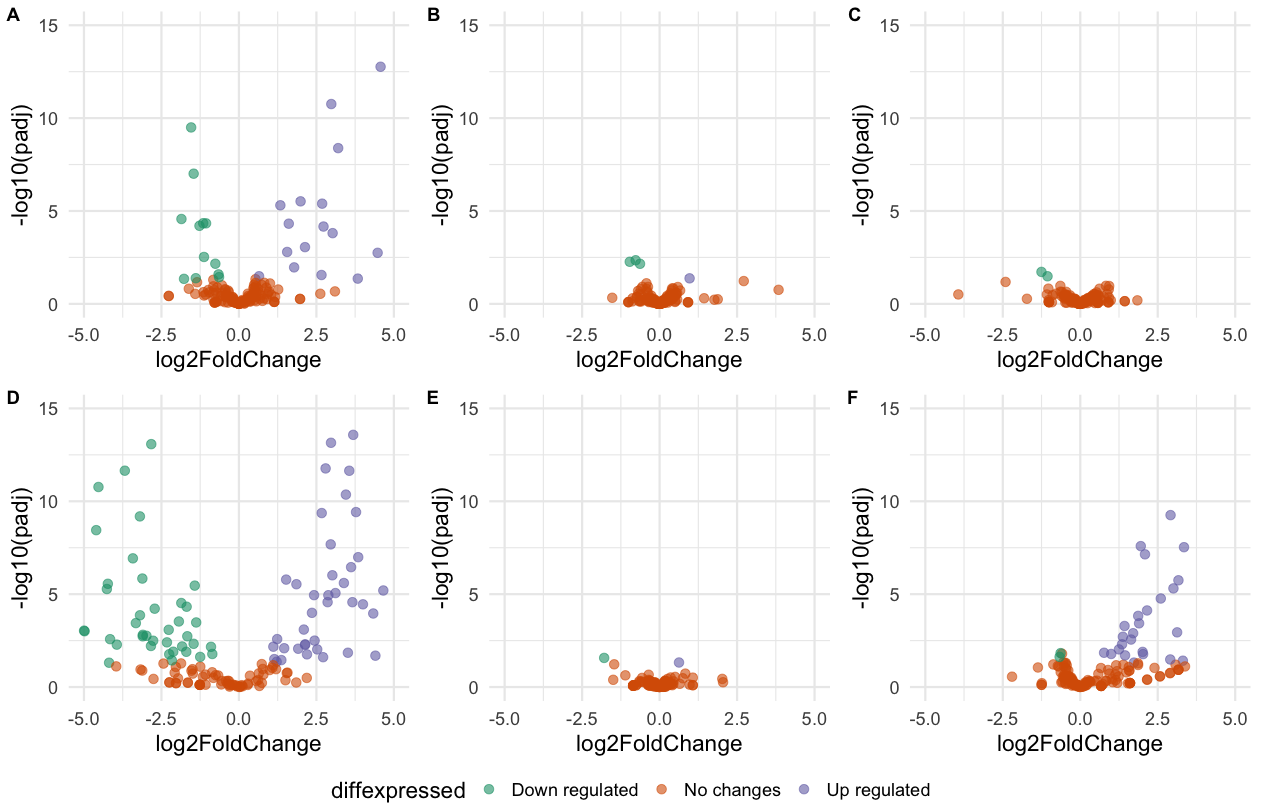

Supplement: Supplementary file 1 — Figure S1 [file MEC-32-904-s001.tiff]

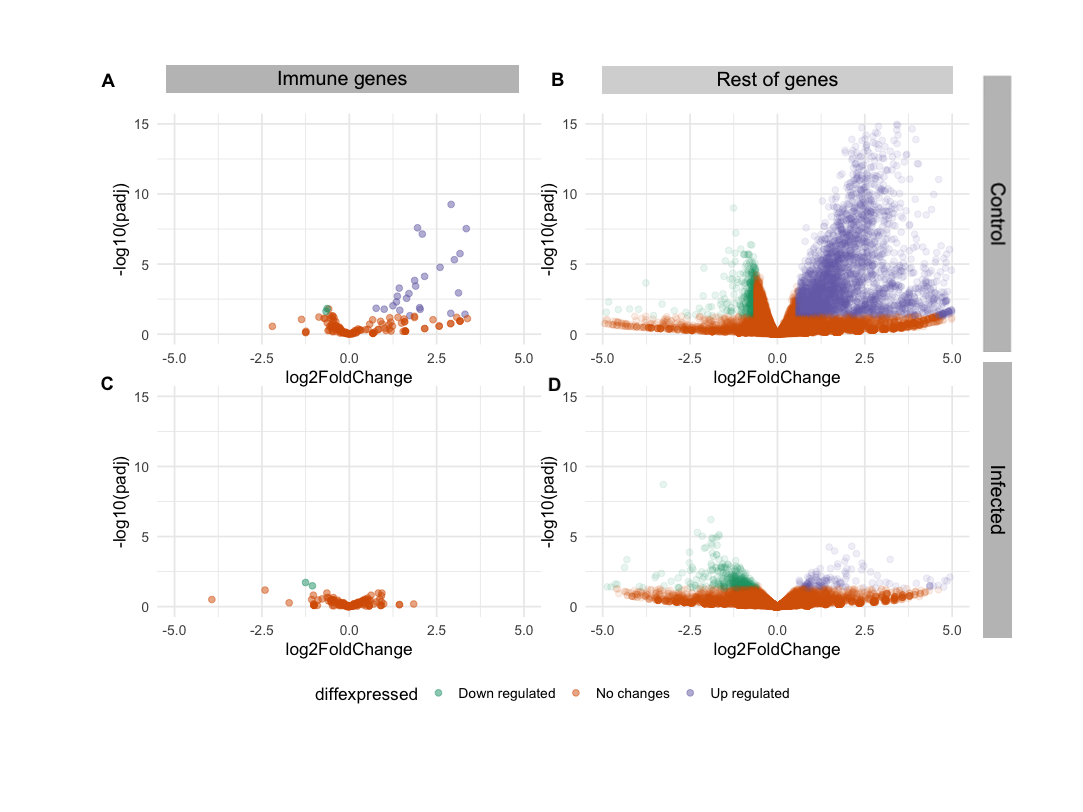

Supplement: Supplementary file 2 — Figure S2 [file MEC-32-904-s003.tiff]
